# Identification and pathogenomic analysis of an *Escherichia coli* strain producing a novel Shiga toxin 2 subtype

**DOI:** 10.1038/s41598-018-25233-x

**Published:** 2018-04-30

**Authors:** Xiangning Bai, Shanshan Fu, Ji Zhang, Ruyue Fan, Yanmei Xu, Hui Sun, Xiaohua He, Jianguo Xu, Yanwen Xiong

**Affiliations:** 1 State Key Laboratory of Infectious Disease Prevention and Control, National Institute for Communicable Disease Control and Prevention, Chinese Center for Disease Control and Prevention, Changping, Beijing China; 2 mEpiLab, New Zealand Food Safety Science & Research Centre, Institute of Veterinary, Animal and Biomedical Sciences, Massey University, Massey, New Zealand; 3 U.S. Department of Agriculture, Agricultural Research Service, Western Regional Research Center, Albany, California USA; 4 Collaborative Innovation Center for Diagnosis and Treatment of Infectious Diseases, Hangzhou, Zhejiang Province China

## Abstract

Shiga toxin (Stx) is the key virulent factor in Shiga toxin-producing *Escherichia coli* (STEC). To date, three Stx1 subtypes and seven Stx2 subtypes have been described in *E. coli*, which differed in receptor preference and toxin potency. Here, we identified a novel Stx2 subtype designated Stx2h in *E. coli* strains isolated from wild marmots in the Qinghai-Tibetan plateau, China. Stx2h shares 91.9% nucleic acid sequence identity and 92.9% amino acid identity to the nearest Stx2 subtype. The expression of Stx2h in type strain STEC299 was inducible by mitomycin C, and culture supernatant from STEC299 was cytotoxic to Vero cells. The Stx2h converting prophage was unique in terms of insertion site and genetic composition. Whole genome-based phylo- and patho-genomic analysis revealed STEC299 was closer to other pathotypes of *E. coli* than STEC, and possesses virulence factors from other pathotypes. Our finding enlarges the pool of Stx2 subtypes and highlights the extraordinary genomic plasticity of *E. coli* strains. As the emergence of new Shiga toxin genotypes and new Stx-producing pathotypes pose a great threat to the public health, Stx2h should be further included in *E. coli* molecular typing, and in epidemiological surveillance of *E. coli* infections.

## Introduction

Shiga toxin-producing *Escherichia coli* (STEC) represents an *E. coli* pathotype producing at least one of Shiga toxins (Stxs), Stx1 and Stx2. STEC has emerged as an important enteric pathogen causing human gastrointestinal disease, ranging from sporadic cases, diarrhea, hemorrhagic colitis (HC), to hemolytic uremic syndrome (HUS) worldwide^1^. Stx, the primary virulence factor of STEC, is an AB5 toxin. The A subunit injures the eukaryotic ribosome, and halts protein synthesis in target cells. The B pentamer binds to the cellular receptor, globotriaosylceramide, Gb3, found primarily on endothelial cells^2^. Stx1 and Stx2, sharing 56% amino acid sequence similarity, are distinguishable based on the inability of antisera to provide cross neutralization^3^. Several Stx1/Stx2 subtypes and variants have been reported^4^. Different Stx subtypes have been reported to vary in receptor preference and toxin potency^5^. The *stx* genes are encoded in heterogeneous lambdoid prophages (Stx phages), which are highly mobile genetic elements in the genome. Stx phages are involved in horizontal genes transfer, thus likely causing the dissemination of *stx* genes among *E. coli* strains. The loss of *stx* genes has also been observed during subculture^6^.

The intestinal tract of ruminants, particularly cattle, have been regarded as the primary reservoir of STECs^7^, which normally does not cause any disease in animals. STEC have also been recovered from other domestic animals, such as sheep, goats, pigs, cats and dogs^8^, as well as wild animals^9,10^. In our previous studies, we depicted the molecular characteristics of STEC in domestic and wild animals, foodstuffs of animal origin, as well as humans in China, which demonstrated dramatically diversity^11–15^. Notably, we systematically investigated the prevalence of STEC in wild animals exclusively residing on the Qinghai-Tibetan plateau, China, an extremely harsh wild environment with elevations between 3500 and 5500 meters above sea level, including yak, pika, antelope and marmot^11,13,14,16^. Our previous studies enlarged the reservoir host range of STECs and further expand the knowledge of their genetic and phenotypic diversity. Genomic analysis revealed that the marmot *E. coli* isolates, including STECs carried a mixed virulence gene pool, and hybrid pathogenic forms were found in different pathotypes of marmot *E. coli* isolates^16^.

Hereafter, we investigated STEC isolates in intestinal contents of the healthy wild *Marmota himalayana*, in the same wild environment, but sampled in different year from our previous study. Here, we emphasized the identification and characterization of a novel Stx2 subtype from marmot STEC strains.

## Results

### Prevelance of STEC in *M. himalayana*

Two hundred marmot intestinal content samples were collected from three sites of Tibet plateau area, and six STEC strains were isolated, giving a culture positive rate of 3%. Among the six s*tx*_2_-positive isolates, four of them (STEC293, STEC294, STEC295, and STEC299) were from marmots sampled at two sites (Table 1). Further characterization showed that these four isolates shared same serotype O102:H18, and sequence type (ST3693) (Table 1). Except for *stx*_2_ gene, the four strains only possessed adhensin related gene *paa* among the seven main STEC virulence-related genes detected by PCR. The other two isolates, STEC296 and STEC297, belonging to the serotype Orough:H8 and O168:H14, had different virulence gene profiles and sequence types (Table 1).

**Table 1:** STEC isolates recovered from intestinal contents of *Marmota himalayana*.

Isolates	Serotype	*stx* subtype	Virulence genes^*^	Sequence type	Site (above m.s. l, latitude/longitude)	Sampling time
STEC293	O102:H18	2h	*paa*	3693	Zhongdaxiang (3599 m, 33°13′/97°01′)	2013-07-29
STEC294	O102:H18	2h	*paa*	3693	Dezhuotan (3025 m, 33°03′/97°11′)	2013-08-02
STEC295	O102:H18	2h	*paa*	3693	Dezhuotan (3025 m, 33°03′/97°11′)	2013-08-03
STEC299	O102:H18	2h	*paa*	3693	Dezhuotan (3025 m, 33°03′/97°11′)	2013-08-02
STEC296	Orough:H8	2a	*ehxA*, *saa*	26	Dedacun (3625 m, 33°06′/97°08′)	2013-08-06
STEC297	O168:H14	2g	*astA*, *saa*	718	Dedacun (3625 m, 33°06′/97°08′)	2013-08-07

*Virulence genes tested include *eae*, *ehxA*, *efa1*, *saa*, *paa*, *toxB*, and *astA*, among which only PCR-positive gene is listed for each isolate.

### Identificaition of a novel Stx2 subtype in STEC strains of Marmot origin

Among six STEC strains isolated in this study, two of them carried *stx*_2_ gene that can be subtyped by the PCR-based method, one carried *stx*_2a_, and the other harbored *stx*_2g_ (Table 1). However, the *stx*_2_ carried by other four isolates (STEC293, STEC294, STEC295, and STEC299) failed to give amplicon using *stx*_2_-subtypes specific primers. The full length *stx*_2_ genes of the four isolates was then amplified. Sequence analysis revealed the *stx*_2_ genes in these four isolates were identical.

**Figure 1:**
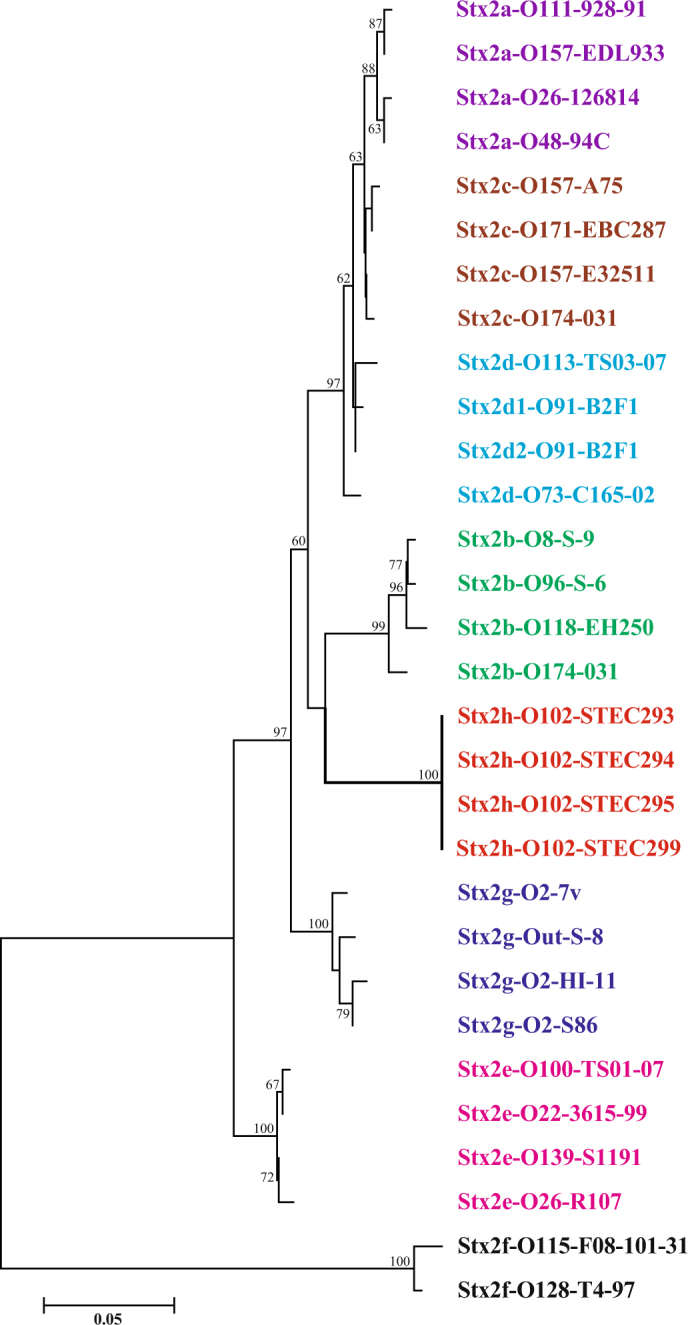
Phylogenetic tree of Stx2 subtypes by the neighbor-joining method. The neighbor-joining tree was inferred from comparison of combined (**A** and **B**) holotoxin amino acid sequences of all Stx2 subtypes. Numbers on the tree indicate bootstrap values calculated for 1000 subsets for branch points >50%. Bar, 0.05 substitutions per site. Stx2 subtypes are indicated by different colors. An extended version of this tree is available as Fig. S2.

**Figure 2:**
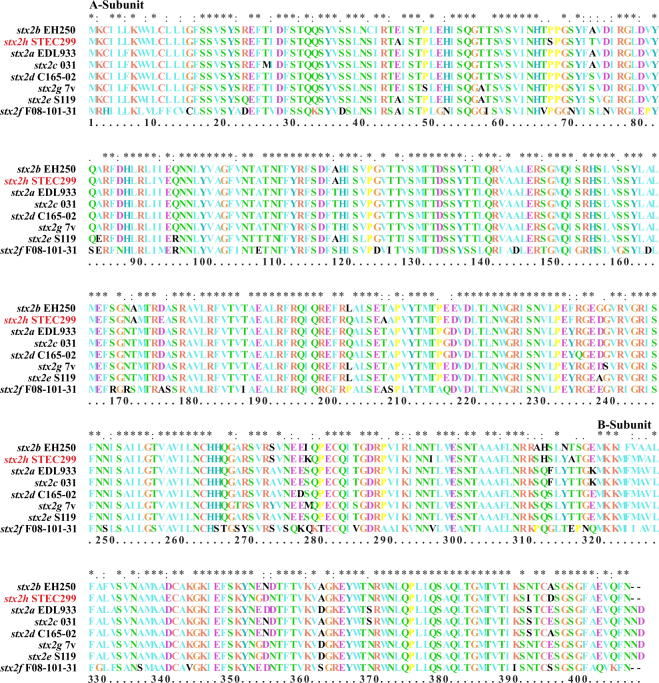
Amino acid alignment of Stx2h (**A** and **B**) subunits against other Stx2 subtypes. Amino acid conserved with all Stx2 sequences are indicated with an asterisk. Differences between sequences are indicated in black letter.

Phylogenetic trees reconstructed using the neighbor-joining algorithm (Fig. 1, see Supplementary Fig. S2 for extended version of this tree), maximum-likelihood and maximum parsimony algorithms shared the same topology and demonstrated that the Stx2 from STEC293, STEC294, STEC295, and STEC299 form a distinct lineage, not clustering with any known Stx2 subtypes and variants. These data suggest these four STEC strains harbor a novel Stx2 subtype. Based on the new nomenclature for Stx, the new Stx subtype was designated Stx2h. STEC299 encoding variant Stx2h-O102-STEC299 was used as type Stx2h strain for further analysis. Comparison of sequences of the *stx*_2h_ subtype and other existing *stx*_2_ subtypes revealed that the nucleic acid sequence of subunit A of the *stx*_2h_ showed a similarity ranging from 69.7 to 92.9% to the previously reported *stx*_2_ subtypes, and 67.2% to 91.3% for subunit B. When comparing sequences of Stx2 holotoxin, the similarity with others ranged from 63.8% to 91.9% in nucleic acid level, and from 71.9% to 92.9% in amino acid level (Table 2). The amino acid alignment for Stx2h-O102-STEC299 holotoxin against other seven subtypes, demonstrated 13 amino acids difference (Fig. 2). The intergenic region between the A and B subunit of *stx*_2h_ contained 12 nucleotides, exhibiting the same intergenic region size with *stx*_2b_, *stx*_2e_, *stx*_2f_ and *stx*_2g_, but display distinct nucleotides composition (CAGGAGTTAAAC) with others (Fig. S1).

**Table 2:** Nucleotide/amino acids identities (%) between *stx*_2h_ and representatives of other seven *stx*_2_ subtypes.

Nucleotide\amino acids	1	2	3	4	5	6	7	8
1.*stx*_2a_		92.6	99.0	97.5	94.1	72.4	97.1	**92.4**
2.*stx*_2b_	91.5		93.1	93.6	91.4	70.9	92.1	**92.6**
3.*stx*_2c_	98.4	91.8		98.0	93.6	71.6	95.1	**92.4**
4.*stx*_2d_	96.8	93.2	97.3		94.1	72.4	95.6	**92.9**
5.*stx*_2e_	91.7	88.7	91.3	91.5		73.6	95.6	**92.6**
6.*stx*_2f_	62.3	62.7	61.8	61.8	69.3		72.9	**71.9**
7.*stx*_2g_	93.9	91.1	92.7	93.7	91.8	64.2		**92.9**
**8**.***stx***_**2h**_	**91.4**	**91.9**	**91.3**	**91.9**	**89.6**	**63.8**	**91.6**	

1. EDL933 (X07865), 2. EH250 (AF043627), 3. 031 (L11079), 4. C165-02 (DQ059012), 5. S1191 (M21534), 6. F08-101-31 (AB472687), 7. 7 v (AY286000), and 8. STEC299 (CP022279).

All four *stx*_2h_-isolates gave an expected band about 150-bp by using the *stx*_2h_-specific PCR, but seven *stx*_2_ subtypes reference strains and a non-O157 STEC collection strains from wild animals in the same sampling region were all negative for *stx*_2h._

### Stx2h is inducible and functional

**Figure 3:**
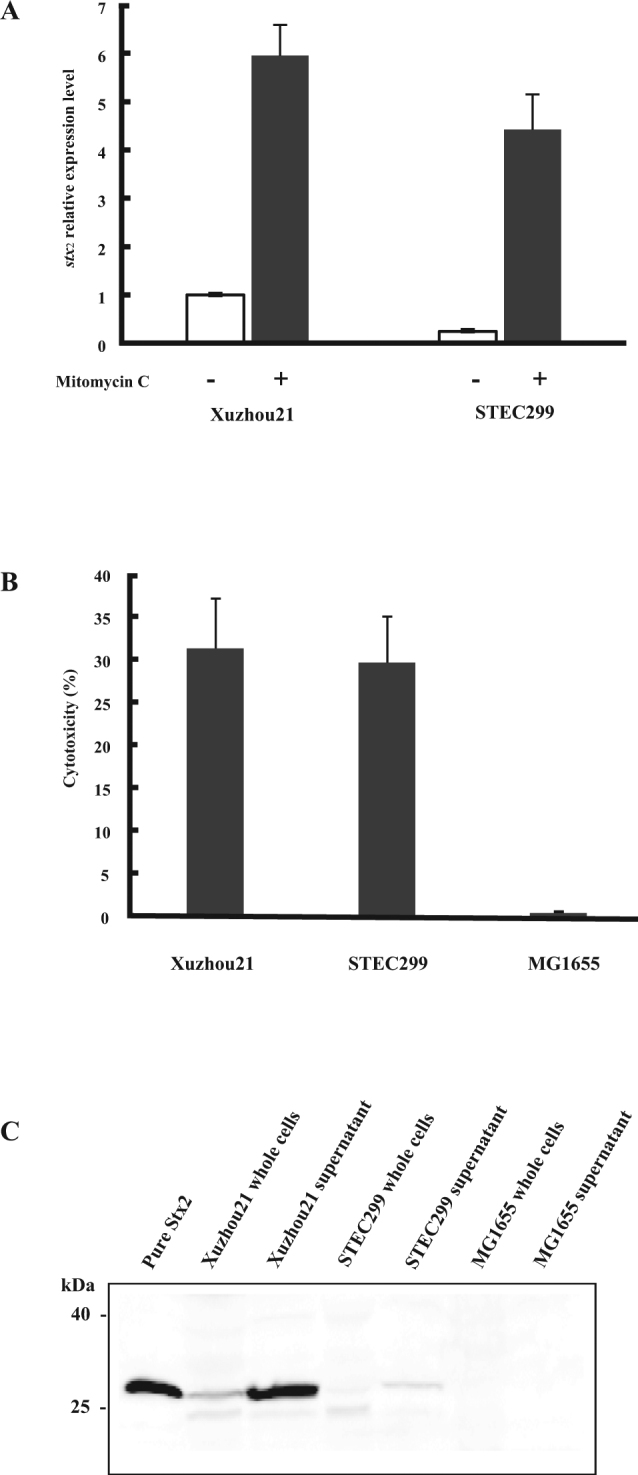
Induction of Stx2h production in STEC299. (**A**) mRNA expression by qRT-PCR. The relative levels of expression under non-induction and induction conditions were relative to the expression level in Xuzhou21 before induction by mitomycin C which was arbitrary set at 1.0. The relative value was averaged from three independent experiments. Error bars represent the standard errors. (**B**) Vero toxicity assay. The cytotoxicity was detected after 24 h incubation exposure to the induced supernatants overnight. Xuzhou21 was used as positive control; MG1655 was used as negative control. (**C**) Western blot assays with an anti-Stx2 rabbit polyclonal antibody on whole cells and supernatants from STEC299 induced with mitomycin C with a final concentration of 0.5 μg/ml. Xuzhou21 was used as positive control.

To determine if Stx2h is inducible, the levels of basal and induced *stx*_2_ expression were determined using real-time RT-PCR. Result showed *stx*_2_ was expressed constitutively (Fig. 3A), while the basal transcription level in STEC299 was lower (4.2 times) than that observed in the O157:H7 outbreak strain Xuzhou21 under non-inducing conditions. Notably, the induced *stx*_2_ expression level is 18.3 times higher than its basal level in STEC299, posting a greater inducing ability than Xuzhou21.The A subunit of novel Stx2h can be recognized against a Stx2 rabbit polyclonal antibody that have been used effectively to recognize all other Stx2 subtypes, while with a lower amount of the protein loaded on the gel (Fig. 3C). Vero cell cytotoxicity assay showed that STEC299 had similar cytotoxicity after 24 hours incubation comparing to the outbreak strain Xuzhou21 (Fig. 3B). These results indicated that *stx*_2h_ expression was inducible and Stx2h had cytotoxicity to Vero cells.

### Stability of *stx*_2_

To evaluate the stability of *stx*_2h_, STEC299 was subcultured daily for two weeks, a sample of overnight growth was detected by a real-time PCR assay for presence of *stx*_2h_. The CT value for each sample remained consistent over the two weeks, indicating that *stx*_2h_ is a relatively stable element within the STEC299 genome.

### Genome features of STEC299

The completed genome sequence of STEC299 consists of a circular chromosome of 4,907,118 bp with a G + C content of 50.7% (Fig. S3A), and two plasmids, pSTEC299-1 of 191,691 bp with a C + G content of 45.5% (Fig. S3B) and pSTEC299-2 (59,190 bp) with a C + G content of 43% (Fig. S3C). The whole genome consists of 5,009 coding DNA sequences (CDSs), 22 rRNA, 94 tRNAs, and 12 prophage/prophage-like elements. We searched the two plasmid sequences in GenBank NR database with BLASTN (accessed 24.07.2017), to identify their closest matches, yet both were distinct from all of the currently published existing sequences, with a highest query coverage of 36% for pSTEC299-1 (*E. coli* strain M18, CP010219.1) and 53% for pSTEC299-2 (*E. coli* plasmid pRPEC180_47, JN935898.1) only.

### Genetic organization of Stx2h prophage

**Figure 4:**
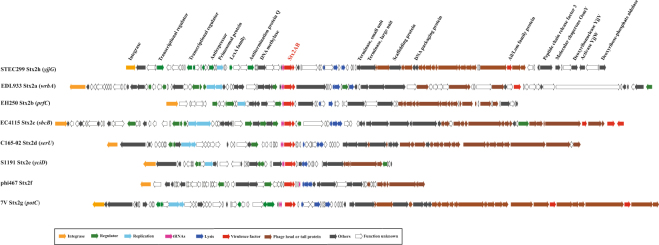
Architecture of Stx2h-converting phages and genomic comparison with other Stx2 phages. The figure shows comprehensive analyses of all Stx2 subtypes converting prophages. Corresponding CDSs are colored as indicated. Integration sites of each phage are presented in parentheses.

Shiga toxin-encoding prophages are highly mobile genetic elements that may result in regulation and horizontal transfer of *stx* genes^17,18^. We further characterized the Stx2 converting prophages including chromosomal insertion site, genetic sequence, and structure. The novel Stx2h converting prophage is 49,713 bp in size in STEC299, and located adjacent to the *yjjG* locus, which found to be a novel insertion site ever reported. In total, 93 CDSs were predicted on the Stx2h prophage, among which, Stx phage-specific genes encoding the integrase, transcriptional regulator, antirepressor, antitermination protein Q, and lysis, were found in STEC299 and other Stx2 subtype reference strains, while 37 were hypothetical proteins or mobile elements with unknown function (Fig. 4). Besides the Shiga toxin gene, virulence-related Ail/Lom family outer membrane protein was detected on the Stx2h prophage, which was also present in the Stx2a, Stx2c and Stx2g prophages (Fig. 4). Comparison with the representatives of seven existing Stx2 subtypes prophages (Stx2a-2g) suggests a high diversity among different Stx subtypes-containing phages, in terms of chromosomal insertion sites, structure, and genetic sequences. Different Stx2 subtype prophages contain extensive non-homologous regions, and sequence dissimilarity was also observed for the same functional protein, such as antitermination Q protein.

### STEC299 exhibited a hybrid virulence genes spectrum

To determine the pathogenic potential of this newly identified Stx2h harboring strain STEC299, the virulence factors were investigated by using a BLAST search against VFDB (http://www.mgc.ac.cn/). Remarkably, except for *stx*_2_, none of STEC main virulence-related factors, like *eae*, *ehxA*, *saa*, *efa1*was identified in STEC299. Instead, many other virulence factors were identified (Table S2), which mainly belonged to five categories: adherence, invasion, autotransporter, iron uptake, and secretion system. The adherence genes, *paa* and Dr family of adhesins encoding gene (*draC*) were found on the large plasmid pSTEC299-1. The type I fimbriae genes (*fimA*, *B*, *C*, *D*, *E*, *F*, *G*, *H*, and *I*), *E. coli* common pilus (ECP)-related genes (*ecpA*, *B*, *C*, *D*, *E*, and *ecpR*), and F1C fimbriae gene (*focC*) were identified on chromosome. The invasion gene *ibeA*, which shared with ExPEC isolates, was detected. In the autotransporter category, enterotoxin gene *espC* and enteroaggregative *E. coli* (EAEC) mucinase gene *pic* were found on plasmid pSTEC299-2. Tsh (*tsh*), another autotransporter prevalent in UPEC and APEC isolates was detected on chromosome. In the iron uptake category, iron-regulated genes (*fyuA*, *irp1*, *irp2*, and *ybtA*, *E*, *P*, *Q*, *S*, *T*, *U*, *X*), hemin uptake-related genes (*chuA*, *S*, *T*, *U*, *W*, *X*, *and Y*) and the iron chelator genes (*entA*, *B*, *C*, *D*, *E*, *F* and *fepA*, *B*, *C*, *D*, *E*) were identified on chromosome. The type II secretion protein genes (*gspD*, *E*, *F*, *G*) were found on the plasmid pSTEC299-2. Notably, the majority of virulence genes in STEC299 are miscellaneous ExPEC virulence determinants, including type I fimbriae genes and invasion gene *ibeA*, which have been regarded as virulence determinants common to ExPEC^19,20^. Additionally, the presence of *fyuA*, *chuA*, and *tsh* identified in STEC299 has been significantly associated with UPEC and NMEC but not diarrhoeagenic *E. coli* (DEC)^21^.

### Phylogenetic position of STEC299

**Figure 5:**
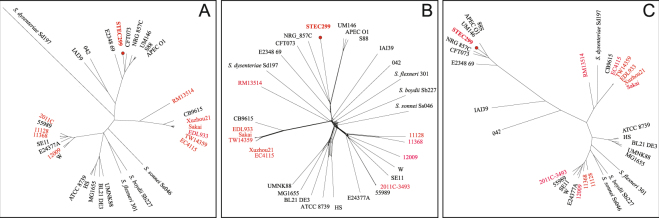
The phylogenetic relationship of the strain STEC299 with the other 32 reference strains. (**A**) ClonalFrame tree of the stains inferred from the concatenated ribosomal protein gene sequences that are single-copy and shared (n = 53) by the 33 strains. Three independent and converged runs were merged and a 95% consensus tree was presented in the final graph. (**B**) Neighbor-net phylogeny generated from wgMLST allele profiles of 2,321 loci that shared by all the strains. The uncertainty and incompatibilities in the dataset were shown as networks. (**C**) Gubbins tree generated with the concatenated sequences of all the shared loci found in wgMLST analysis. STEC strains are highlighted in red on the three different trees.

The phylogenetic position of STEC299 among a diverse collection of *E. coli* and *Shigella spp*. genome sequences comprised of representatives of all major pathotypes was assessed using ribosomal multilocus sequence analysis, whole-genome multilocus sequence typing (wgMLST) and whole-genome phylogenetic analysis. In total 53 ribosomal protein gene sequences were extracted from the annotated whole-genome sequence of the 33 strains. Ribosomal protein L36 and L31 type B gene was excluded from the analysis because of the presence of paralogues in some genomes. A ClonalFrame tree (Fig. 5A) was inferred from the 53 concatenated ribosomal protein gene sequences that are single-copy and shared by the 33 strains, which revealed that the novel Stx2h converting strain STEC299 formed a remote cluster with all 10 reference STEC genomes, while grouped close with strains representing EPEC, UPEC, NMEC, APEC, AIEC. Similarly, neighbor-net phylogeny (Fig. 5B) and Gubbins tree (Fig. 5C) generated with the concatenated sequences of all the 2,321 shared loci found in wgMLST analysis were also consistent with this finding. Our study indicates that the Stx2h-containing strain STEC299 is phylogenetically closer to other pathotypes of *E. coli* than STEC, thus we propose that STEC299 might evolve from other pathotypes into STEC by acquiring Stx prophage and other virulence factors.

## Discussion

Shiga toxins (Stx1 and Stx2) are key virulence factors in the pathogenesis of gastroenteritis, HC and HUS, caused by STEC and other Stx-producing bacteria, including *Citrobacter freundii*, *Enterobacter cloacae*, *Acinetobacter haemolyticus*, *Aeromonas sp*., and *Escherichia albertii*^22–26^. A previous study to standardize Stx nomenclature proposed three Stx1 subtypes (Stx1a, Stx1c, and Stx1d) and seven Stx2 subtypes (Stx2a to 2g), based on phylogenetic analysis of Stx holotoxin sequences^4^. Stx2 was reported to be more often correlated with severe clinical outcomes and development of HUS than Stx1^27^. Further, different Stx2 subtypes are associated with varied clinical symptoms. Strains producing Stx2a, Stx2c, or Stx2d subtype which display close sequences relatedness, are more often correlated with development of HC and HUS^27–29^, while those producing other (more distantly related) subtypes (Stx2b and Stx2e to Stx2g) are primarily related to a milder course of disease.

Stxs normally reside in bacteriophages, where horizontal gene transfer could lead to emergence of new Stx-subtypes/variants or Stx-producing pathogens^18^. In a recent study, a novel Stx1 subtype, Stx1e, was identified from an *Enterobacter cloacae* strain^30^. The emerging of the new *stx* subtypes/variants, and new Stx-producing pathotypes pose a great threaten to the public health. Here, we reported a novel Shiga toxin 2 subtype, named Stx2h produced by *E. coli* O102:H18 strains from marmots in Qinghai–Tibet plateau of China. Stx2h shows high induced level of *stx*_2_ expression, and cytotoxicity to Vero cell, and the reactivity with anti-Stx2 antibody, posing diagnostic challenge for the emerging of new Stx subtypes/variants. Remarbly, the novel Stx2h subtype occurred at an unexpectedly high rate of 66.7% (4 of 6) in marmot STEC strains, but was not detected in any of other animal-derived or human STEC strains we investigated in different regions of China so far. The absence of Stx2h in other animal in the same enviroment suggests that it may be a recently emerged subtype that has not yet extensively spread among animals, or it could be limited to a specific host or ecosystem. The occurrence of the new Stx2h subtype from marmots enlarges the pool of Stx2 subtypes and add further information to the global epidemiological picture of STEC strains. Future work should bring into light if the novel Stx2h subtype is specific for STEC strains adapted to marmot or wild animal on the Qinghai-Tibetan plateau.

Pathogenic *E. coli* can be categorized into different pathotypes based on the presence of specific virulence markers. The *stx* genes specific for STEC reside in the genome of heterogeneous lambdoid prophages, Stx-converting bacteriophages^31^, which could infect various bacterial hosts wider than expected^18^. The potential genetic combinations due to gene transfer may result in hybrid pathotype strains. Stx2-phages can infect and lysogenize almost all known pathotypes of *E. coli*, including both diarrheagenic *E. coli* (DEC) and extraintestinal pathogenic *E. coli* (ExPEC)^17^. The emergence of novel hybrid form of STEC and other *E. coli* pathotypes might result in more severe disease. For instance, the STEC/enteroaggregative *E. coli* (EAEC) hybrid strain O104:H4 caused a large outbreak with numerous HUS cases in Germany in 2011^32^. STEC/enterotoxigenic *E. coli* (ETEC) hybrid strains have been recovered from various sources and correlated with diarrheal disease and even HUS in humans^33,34^. A recent report described an STEC/ExPEC hybrid that caused HUS and bacteremia^35^. STEC/UPEC hybrid strains have also been identified from hospital patients^20,36^. Our study reveals the presence of virulence factors from multiple pathotypes of *E. coli* in the Stx2h converting strain STEC299, including type I fimbriae genes, *ibeA*, *chuA*, *fyuA*, and *tsh*^19–21,37^. The gene *pic*, originally identified in the EAEC prototype strain 042^38^, was present frequently among UPEC strains, with a positive rate of 13% reported by Abe *et al*.^39^. The autotransporter gene *tsh* in *E. coli* strains are associated with acute pyelonephritis, and are expressed during urinary tract infection^40^. Considering the severe disease caused by hybrid pathotypes, the pathogenic potential of STEC299 should not be neglected and calls for considerable attention. Further, the combined virulence traits of STEC299 is in accordance with our previous findings that most of the marmot *E. coli* strains exhibited hybrid forms carrying virulence markers from various pathotypes^16^, indicating that marmot *E. coli* strains exhibit a marked genome plasticity. Future work should aid to ascertain if the *E. coli* strains from marmots show more tendency to represent hybrid genotypes.

Phylogenies inferred from whole genome comparision clearly underlines that STEC299 are phylogenetically closer to other pathotypes of *E. coli* than STEC group, thus we propose that marmot strain STEC299 may evolved from other pathotypes by horizontal gene transfer and gaining Stx2 phage, which is supported by view that pathogenic *E. coli* was evolved from non-pathogenic *E. coli* through horizontal transfer of virulence genes, resulting in mixed pathotypes with enhanced pathogenicity^41^. However, there are some limitations in genomic analysis, as the number of ExPEC and other pathotypes used for genome comparison is small due to the limited completed reference genomes available, further study are needed to clarify the evolution pattern. Moreover, reference genomes used for comparison are mostly from human-derived strains, there might be a possibility that strains are phylogenetically divergent based on the host origins, thus a various collection of strains are further needed for better understand the phylogenetic placement.

In conclusion, we report the discovery of a novel Shiga toxin 2 subtype from marmot *E. coli* strains, and enlarges the pool of Stx2 subtypes. Our study shows the new Stx2h converting strain STEC299 is a heteropathogenic strain, which is closer to other pathotypes of *E. coli* in terms of both phylogenies and virulence gene spectrum. As the emergence of new Stx subtypes and Stx-producing pathotypes has represented a serious problem with the tendency to cause more severe disease, the novel Stx2h should be further included in molecular typing of *E. coli* strain, and in epidemiological surveillance of *E. coli* infections.

## Methods

### Ethics statement

The Marmots (*M. himalayana*) were sampled as part of the animal plague surveillance program conducted in Yushu Tibetan autonomous prefecture, Qinghai province. The sampling was performed in accordance with the protocol for national plague surveillance program in animals. The study has been reviewed and approved by the ethic committee of National Institute for Communicable Diseases Control and Prevention, China CDC.

### Sampling and strain isolation

Of a total of 200 Marmots sampled between July and August 2013, 51 were from Zhongdaxiang (with an altitude of 3599.6 m above sea level (a.s.l)), 120 from Dezhuotan (3025 m a.s.l), and 29 from Dedacun (3625.6 m a.s.l), respectively. The Marmots were captured by cages in the field and sampled in the laboratory of local Centre for Disease Control (CDC). The intestinal contents were collected in 2 ml sterile tubes containing Luria-Bertani (LB) medium in 30% glycerol, which were stored at −20 °C immediately and transported to the laboratory in the National Institute for Communicable Disease Control and Prevention in Beijing. Strains were isolated and confirmed to be STEC by the methods we previously described^11,13^. Briefly, enriched samples in *E. coli* broth (Land Bridge, Beijing, China) were examined by PCR for the presence of *stx* genes with primers stx1F/Stx1R and Stx2F/Stx2R respectively^13^. PCR-positive enrichments were then streaked onto CHROMagar^TM^ ECC agar (CHROMagar, Paris, France), and MacConkey agar (Oxoid, Hampshire, UK). Colonies resembling *E. coli* were picked and tested for *stx* genes by single colony duplex PCR assay. Serotyping, detection of main STEC-related virulence factors (*stx*, *eae*, *ehxA*, *efa1*, *saa*, *paa*, *toxB*, *and astA*), and multilocus sequence typing (MLST) were conducted as we previously described^11,13^.

### Stx subtyping based on phylogenetic analysis

*stx* subtypes of STEC isolates were determined by the PCR-based subtyping method^4^. For strains that failed to be detected by the *stx*_2_ subtype-specific primers, the completed *stx*_2_ gene was amplified as described previously^11,42^, then cloned into vector pMD18-T and transformed into *E. coli* JM109 (Takara, Dalian, China). About 10 transformants were selected for sequencing to discern multiple *stx*_2_ subtypes in a PCR product. The 93 representative reference nucleotide sequences of the full *stx*_2_ operon of *stx*_2_ subtypes and variants (*stx*_2a_-*stx*_2g_) were downloaded from GenBank as previously described^4^. The amino acid sequences for the combined A and B holotoxin were translated from the open reading frames. The full nucleotide and amino acid sequences, including A and B subunits, the intergenic regions, were aligned and compared by using Clustal Omega to evaluate the differences between *stx*_2_ sequences. Phylogenetic trees based on the holotoxin amino acid sequences were reconstructed with three algorithms, neighbor-joining, maximum likelihood and maximum parsimony, using MEGA 7 software (www.megasoftware.net)^43^, and the stability of the groupings was estimated by bootstrap analysis (1000 replications). Genetic distances were calculated by the maximum composite likelihood method.

### Developing a specific PCR to detect and subtype *stx*_2h_

The *stx*_2h_ and reference nucleotide sequences of *stx*_2a_-*stx*_2g_ were aligned. Subtype-conserved areas were searched to develop a pair of *stx*_2h_-specific primers Stx2h-F (5′-AGATCTCATTCTTTATATG-3′) and Stx2h-R (5′-TCCCCATTATATTTAGAG-3′). The PCR cycling conditions were as follows: an initial denaturation at 94 °C for 5 minutes followed by 30 cycles, each consisting of 30 seconds at 94 °C, 30 seconds at 51 °C and 30 seconds at 72 °C, and a final elongation at 72 °C for 5 minutes using Premix *Taq*™ ((TaKaRa, Japan). The expected PCR product is 149-bp. Seven *stx*_2_ reference strains and a non-STEC collection from yak, marmot, pika, antelope, cattle, goat, pig, food, diarrheal patients and healthy carriers reported previously^14^ were tested by this subtyping protocol.

### Determination of *stx*_2_ transcription by real-time reverse-transcription (RT)-PCR

Strains were grown in Luria-Bertani medium at 37 °C with shaking to an OD_600_ of 0.6., Mitomycin C (BBI, USA) was added to a final concentration of 0.5 μg/ml and incubated for three hours to induce the Stx2 phage. Total RNA was extracted with RNeasy Mini Kit (Qiagen, Germany). Real-time RT-PCR was performed with the Rotor-Gene Q system (Qiagen, Germany) using a One Step SYBRH PrimeScript^TM^ RT-PCR kit (TaKaRa, Japan), according to the manufacturer’s instructions. The RT-PCR profile was as follows: 42 °C for 10 minutes, 95 °C for 10 seconds, and 40 cycles of 95 °C for 15 seconds, 60 °C for 1 minute. Primers stx2F (CAACGGACAGCAGTTATACCACTCT) and stx2R (TTAACGCCAGATATGATGAAACCA) allowed amplification of an *stx*_2_ fragment. Primers gapA-F (TATGACTGGTCCGTCTAAAGACAA) and gapA-R (GGTTTTCTGAGTAGCGGTAGTAGC) allowed amplification of an *gapA* fragment. Expression levels of house-keeping gene *gapA* (D-glyceraldehyde-3-phosphate dehydrogenase) were used as endogenous control within each sample. The relative level of *stx*_2_ expression was calculated using the 2^−ΔΔCT^ method^44^ and the expression in *E. coli* O157:H7 strain Xuzhou21 under non-inducing condition was arbitrary set at 1.0. The experiment was performed in triplicate for each isolate.

### Detection of Stx2 production by Western blot

Western blots were conducted as described^45^. Briefly, the culture was induced with mitomycin C and incubated overnight. The supernatants and cells were harvested and separated by SDS-PAGE. After PAGE, the proteins were transferred to an Immobilon^®^ PVDF membrane (pore size, 0.45 µm; Merck, Germany). Stx2 rabbit polyclonal antibody was diluted to 1 μg/ml in PBS buffer and incubated for 3 hours at room temperature, and then washed three times in PBST. Goat anti-Rabbit IgG (H + L) (IRDye^®^ 800CW) at a 1/20,000 dilution was incubated for 2 h at RT. The blots were washed four more times with PBST, and then visualized with a 5 minutes exposure with a LI-COR Odyssey scanner (LI-COR Biosciences, USA).

### Vero cell cytotoxicity

The cell-free supernatants were used for Vero cytotoxicity assay. Briefly, Vero cells were maintained in tissue culture flasks in DMEM (Difco, USA) supplemented with 10% fetal calf serum at 37 °C in an atmosphere of 5% CO_2_ for 24 h. Filtrates were added in triplicate to Vero cells (10^4^ cells per well) in 96-well tissue culture plates, then incubated at 37 °C in a 5% CO_2_ atmosphere. After 24 h, the release of the cytoplasmic lactate dehydrogenase (LDH) was detected using the Cytotox96 kit (Promega, USA) according to the manufacturer’s instructions. The percentage of cytotoxicity was determined as (experimental release-spontaneous release)/(maximum release-spontaneous release) × 100. The spontaneous release was the amount of LDH activity in the supernatant of uninfected cells, the maximum release was that when cells were lysed with the lysis buffer. *E. coli* Xuzhou21 and *E. coli* MG1655 were used as control.

### *stx*_2_ stability

The stability of *stx*_2_ was evaluated as previously described^30^. Briefly, subcultures of bacteria were prepared for 14 consecutive days. Nucleic acid extracts from the consecutive subcultures were tested using the Rotor-Gene Q system (Qiagen, Germany). The primer Stx2F (GGGCAGTTATTTTGCTGTGGA), Stx2R (GAAAGTATTTGTTGCCGTATTAACGA) and probe Stx2-P FAM-ATGTCTATCAGGCGCGTTTTGACCATCTT-BHQ1were used for *stx*_2_detection as described previously^46^. The reaction mixture consisted of 5 μL of DNA extract, 15 μL of 2 × Premix Ex Tap (Probe qPCR) (TaKaRa, Japan), 1 μL of Stx2-F primer (50 μM), 1 μL of Stx2-R primer (50 μM), 0.5 μL of FAM-labeled probe of *stx*_2_(5 μM). Cycling conditions used for real-time PCR amplification were as follows: 50 °C for 2 min, 95 °C for 10 min, and 40 cycles at 95 °C for 15 s and60 °C for 1 min.

### DNA isolation and whole-genome sequencing

Genomic DNA was isolated from an overnight culture using the Wizard Genomic DNA purification kit (Promega, USA) according to the manufacturer’s instructions. The complete genome was sequenced by single molecule, real-time (SMRT) technology using the Pacific Biosciences (PacBio) sequencing platform^47^. The data were assembled to generate one circular genome without gaps by using SMRT Analysis 2.3.0^48^. The protein-coding sequences (CDSs), tRNAs and rRNAs were predicted using GeneMarkS^49^. The prophages were predicated by the PHAge Search Tool (PHAST)^50^. The virulence factors were predicted through the BLAST tool of NCBI and by using the virulence factor database (VFDB; http://www.mgc.ac.cn/)^51^.

### Stx containing prophage sequence analysis

The sequence of the Stx converting phage was extracted from the complete genome by using the PHASTER (http://phaster.ca/)^52^, the genome of Stx converting phage was reannotated using the RAST server (http://rast.nmpdr.org/)^53^, and then manually verified and corrected. Functional annotation of selected CDSs was performed based on the results of homology searches against the public nonredundant protein database (http://www.ncbi.nlm.nih.gov/) by using BLASTP. The gene adjacent to the integrase was designated as the phage insertion site^54^. The full Stx2 phage sequence of the STEC299 was compared in detail to representative Stx2 converting phages and visualized using perl script. The Stx2 phage sequences of the reference strains used in the current study were kindly provided by Dr. David A. Rasko, University of Maryland School of Medicine^54^.

### Phylogenomic Analysis

To generate a robust, high-resolution phylogenomic tree depicting position of the novel Stx2 converting strain STEC299, the genome were compared with 32 *E. coli*/*Shigella spp*. completed genomes comprised of representatives of all the major pathotypes (Table S1) by using two strategies: ribosomal protein gene sequence analysis (rMLST)^55^ and whole-genome multilocus typing (wgMLST). The ribosomal protein subunites (*rps*) gene sequences were extracted from the annotated whole-genome sequence of the 33 strains. Three independent runs were then carried out with ClonalFrame (version 1.2) on the extracted *rp*s gene sequences and the outputs of the analyses were converged and merged to generate a 95% consensus tree^56^. For wgMLST analysis, the completed whole-genome sequence of strain EDL933was used as reference to perform an *ad hoc* wgMLST analysis using Genome Profiler version 2.0^57^. The relationship of the strains was further analyzed with Splits Tree 4^58^. The whole-genome phylogeny was inferred from the concatenated sequences of the loci shared by the 33 whole-genome sequences, which was found in the wgMLST analysis. All the regions with elevated densities of base substitutions were eliminated and phylogenetic relationship were generated by Gubbins^59^.

### Accession numbers

The complete genome sequences of STEC299 are available at GenBank under the accession numbers: STEC299 chromosome (CP022279), plasmid pSTEC299-1 (CP022280), and plasmid pSTEC299-2 (CP022281).

## Electronic supplementary material

Supplementary Material
