# Supplementary material for: Identification and pathogenomic analysis of an Escherichia coli strain producing a novel Shiga toxin 2 subtype
Source: Sci Rep. 2018 Apr 30;8:6756. doi: 10.1038/s41598-018-25233-x (PMC5928088; doi:10.1038/s41598-018-25233-x)
Supplement: Supplementary file 1 — Supplementary Material [file 41598_2018_25233_MOESM1_ESM.pdf]

Supplementary Information

Identification and Pathogenomic Analysis of an *Escherichia coli* Strain Producing a Novel Shiga Toxin 2 Subtype

Running title: A novel Subtype of Shiga Toxin 2

Xiangning Bai [1†], Shanshan Fu [1†], Ji Zhang [2†], Ruyue Fan [1], Yanmei Xu [1], Hui Sun [1], Xiaohua He [3], Jianguo Xu[1,4], and Yanwen Xiong [1,4*]

1. State Key Laboratory of Infectious Disease Prevention and Control, National Institute for Communicable Disease Control and Prevention, Chinese Center for Disease Control and Prevention, Changping, Beijing, China;

2.mEpiLab, Institute of Veterinary, Animal and Biomedical Sciences, Massey University, New Zealand;

3. Western Regional Research Center, U.S. Department of Agriculture, Agricultural Research Service, Albany, California, USA;

4. Collaborative Innovation Center for Diagnosis and Treatment of Infectious Diseases, Hangzhou, Zhejiang Province, China

† These authors contributed equally to this work.

* Corresponding author. Tel.: +8610 58900749; Fax: +8610 58900748

E-mail address: xiongyanwen@icdc.cn

Table S1. *E. coli/Shigella* genomes used in whole genome analysis.

Isolate	Pathovar	Accession number
EDL933	STEC [a]	NZ_CP008957.1
Sakai	STEC [a]	NC_002695.1
Xuzhou21	STEC [a]	NC_017906.1
EC4115	STEC [a]	NC_011353
TW14359	STEC [a]	NC_013008
2011C-3493	STEC [b]	NC_018658.1
12009	STEC [c]	NC_013353.1
11368	STEC [d]	NC_013361.1
RM13514	STEC [e]	CP006027.1
11128	STEC [f]	NC_013364.1
NRG 857C	AIEC	NC_017634
UM146	AIEC	NC_017632
042	EAEC	NC_017626
55989	EAEC	CU928145
CB9615	EPEC	NC_013941
E2348/69	EPEC	NC_011601
UMNK88	ETEC	NC_017641
E24377A	ETEC	NC_009801
IAI39	UPEC	NC_011750
CFT073	UPEC	NC_004431
S88	NMEC	NC_011742
APEC O1	APEC	NC_008563
SE11	Commensal	NC_011415
HS	Commensal	NC_009800
MG1655	K12	NC_000913
W	Lab	NC_017635
ATCC 8739	Lab	NC_010468
BL21(DE3)	Lab	NC_012971
Shigella sonnei Ss046	*Shigella*	NC_007384
S. dysenteriae Sd197	*Shigella*	NC_007606
S. flexneri 301	*Shigella*	NC_004337
*S. boydii*227	*Shigella*	NC_007613

[a]O157:H, [b]O104:H4, [c]O103:H2, [d]O26:H11, [e]O145:H28, [f]O111:H-

Table S2. Virulence traits of strain STEC299

***** C, virulence genes on chromosome; p1, genes on plasmid pSTEC299-1; p2, genes on plasmid pSTEC299-2.

Gene	Description	Presence/Postion*
stx	Shiga-toxin 1	Negative
stx2	Shiga-toxin 2	Positive/C
eae	Intimin (adhesin)	Negative
ehxA	Enterohaemolysin A	Negative
saa	Shiga-toxin-producing *E. coli* autoagglutinationadhesin	Negative
efa1	Enterohaemorragic *E. coli* factor for adherence	Negative
fuyA	Yersiniabactinsiderophore (receptor)	Positive/C
chuA	hemin uptake-related factor	Positive/C
ibeA	Endothelial invasion	Positive/C
tsh	Temperature-sensitive haemagglutinin	Positive/C
fimA	Type I fimbriae	Positive/C
yfcV	Fimbrial protein	negative
paa	Adherence	p1
gsp	Type II secretion protein	p1
pic	Pic involved in the intestinal colonization	p2
draC	Dr family of adhesins	p1
focC	F1C fimbriae	C
ecpA	*E. coli* common pilus	C
draC	Dr family of adhesins	p1
irp1	Yersiniabactin biosynthetic protein	C
irp2	Yersiniabactin biosynthetic protein	C
entA	Iron chelator	C
espC	Enterotoxin gene	p1
ecpA	*E. coli* common pilus	C

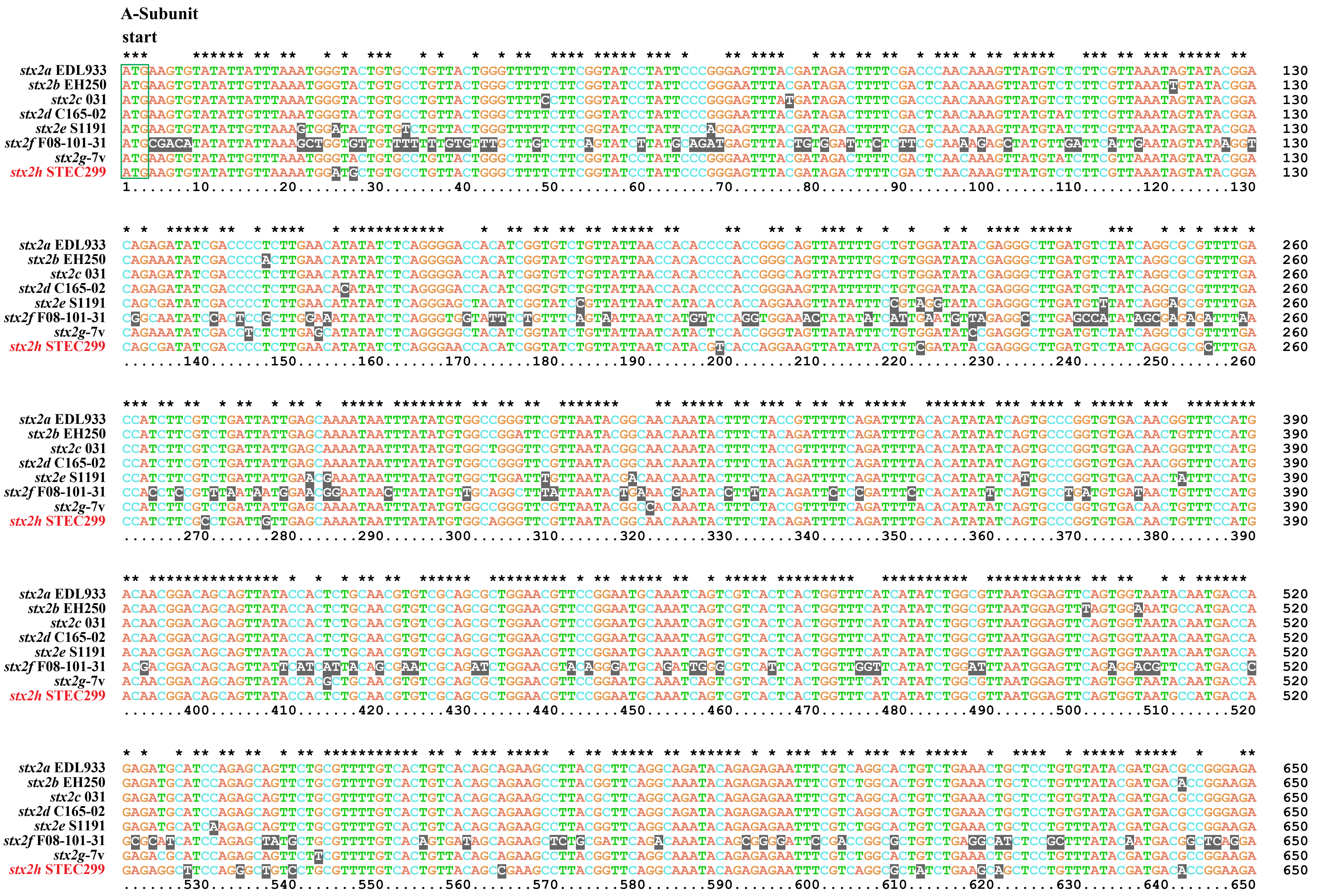
A-Subunit
start
stx2a EDL933
stx2b EH250
stx2c 031
stx2d C165-02
stx2e S1191
stx2f F08-101-31
stx2g-7v
stx2h STEC299

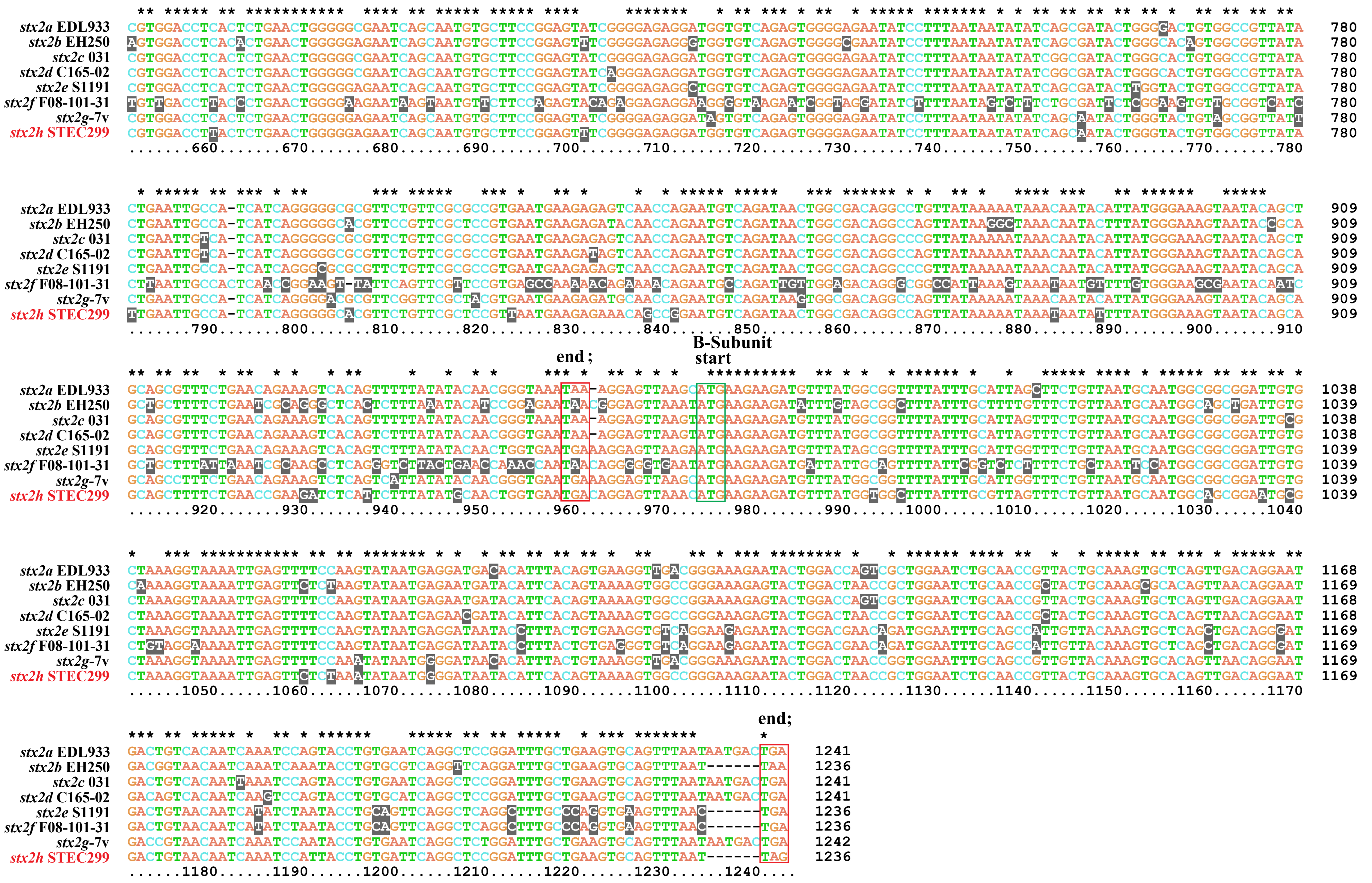

Figure S1. Nucleotide comparison of *stx*2h with other *stx*2 subtypes.
Nucleotide conserved with the *stx*2 sequences are indicated with an asterisk. Shaded letters represent nucleotide differences between the sequences of different *stx*2 subtypes. Nucleotides in green and red boxes indicate start and stop codon respectively for subunit A and B.

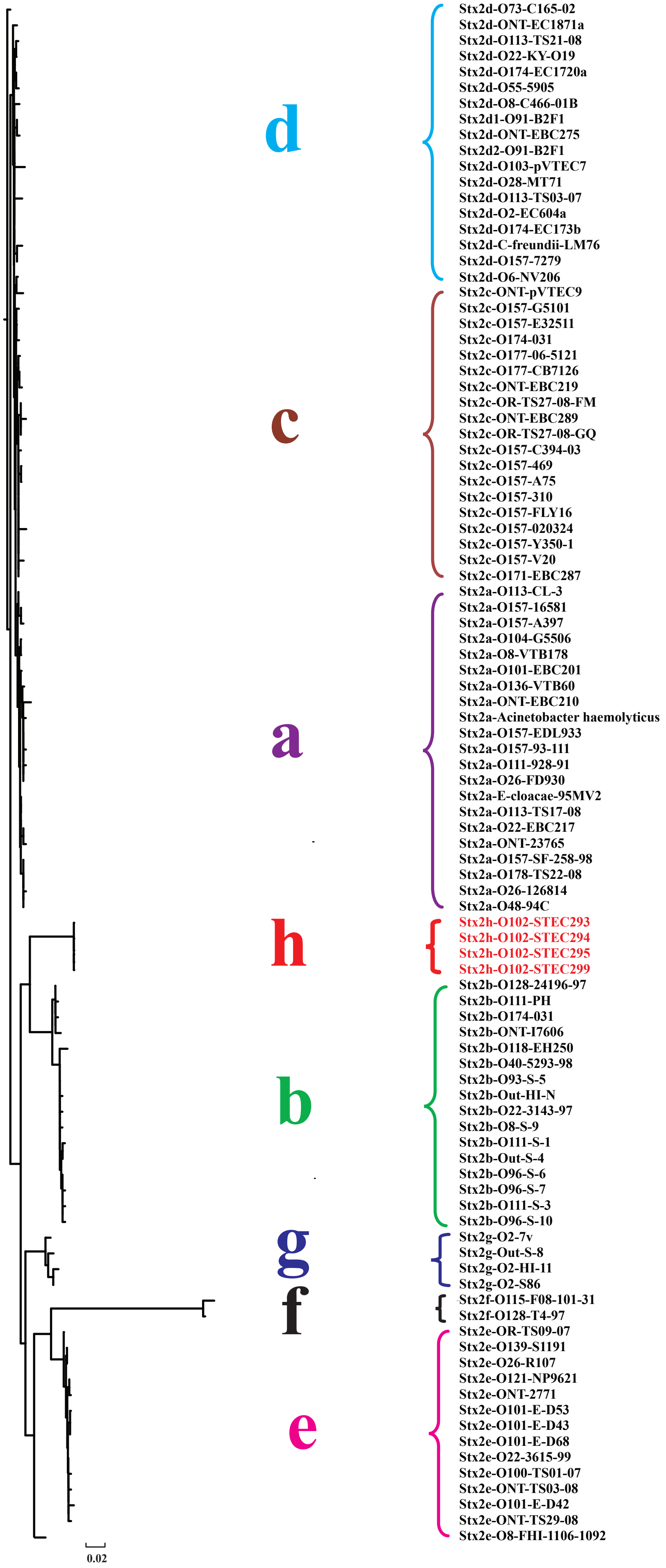

Figure S2. Phylogenetic analysis of Stx2 subtypes and variants by the neighbor-joining method
The neighbor-joining tree was inferred from comparison of combined A and B holotoxin amino acid sequences of Stx2h and 93 representatives of other known Stx2 subtypes and variants. Bar, 0.02 substitutions per site. Stx2 subtypes are indicated by different colors.

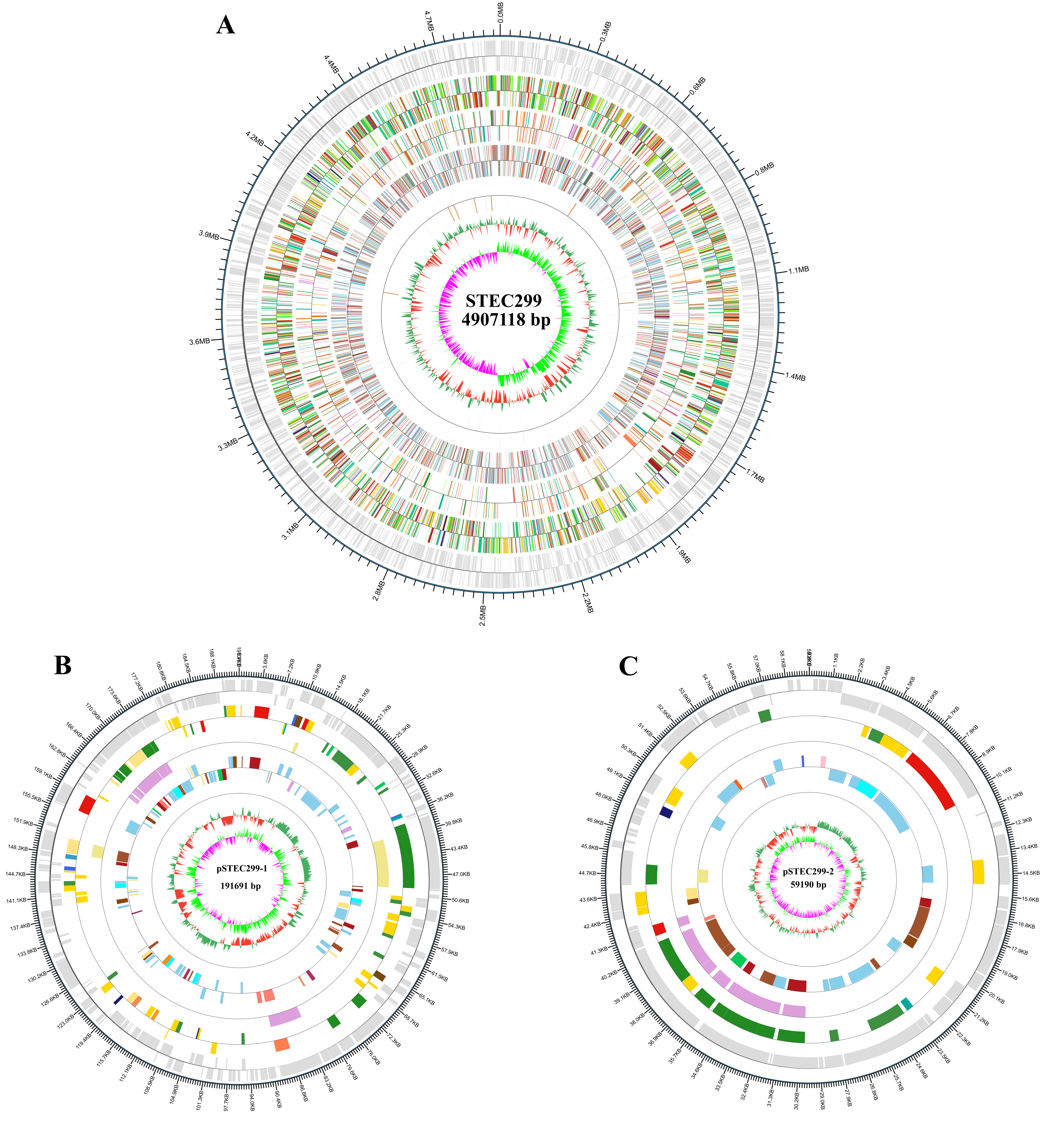

Figure S3. Genome features of STEC299.

Circular maps of the chromosome (A), and the two plasmids, pSTEC299-1 (B) and pSTEC299-2 (C). For both chromosome and plasmids, from outer circle to inner circle, each represents CDS (circle 1 and 2), COG annotation (circle 3 and 4), KEGG annotation (circle 5 and 6), GO annotation (circle 7 and 8), ncRNA (circle 9 and 10), GC Content (circle 11), GC Skew (circle 12). The two circles in one parentheses contains positive and negative strands respectively.
